# Reassessing the Value of Contrast-Enhanced Ultrasonography in Differential Diagnosis of Cervical Tuberculous Lymphadenitis and Lymph Node Metastasis of Papillary Thyroid Carcinoma

**DOI:** 10.3389/fonc.2021.694449

**Published:** 2021-10-14

**Authors:** Xu Zhang, Lingling Wang, Na Feng, Tu Ni, Wei Tang

**Affiliations:** Department of Ultrasound, Affiliated Hangzhou Chest Hospital, Zhejiang University School of Medicine, Hangzhou, China

**Keywords:** contrast-enhanced ultrasonography, lymph nodes, metastasis, papillary thyroid cancer, tuberculous

## Abstract

**Aim:**

We aimed to evaluate the ability of contrast-enhanced ultrasonography (CEUS) to perform differential diagnosis of cervical tuberculous lymphadenitis and lymph node metastasis from papillary thyroid cancer (PTC).

**Methods:**

We analyzed 102 enlarged cervical lymph nodes as diagnosed by conventional ultrasound (US) and CEUS. The US and CEUS enhancement pattern and the time intensity curve (TIC) of the metastatic lymph nodes or tuberculous lymph nodes were compared following standard pathological protocols. The TIC included peak time (TTP), peak intensity (PI), and area under the gamma curve (AUC).

**Results:**

Pathological results demonstrated that 48 out of the 102 enlarged lymph nodes were lymph node metastasis from PTC, while 54 were tuberculous lymphadenitis. There was statistically significant differences in hyperechoic islands, pulse-like enhancement, and asynchronous enhancement between tuberculous lymphadenitis and lymph node metastasis (*P* < 0.05), but their diagnostic sensitivity and specificity were unsatisfactory. In addition, our data did not show statistically significant difference in enhancement direction, enlarged range on CEUS, and perfusion defect (*P* > 0.05). Similarly, quantitative parameters such as PI, TTP, and AUC did not yield significant differences between the groups.

**Conclusion:**

Taken together, the present results demonstrate that CEUS can provide valuable information on lymph node blood flow, which can be used to identify tuberculous lymphadenitis and lymph node metastasis of PTC.

## Introduction

Lymph node metastasis and tuberculous lymphadenitis are the leading causes of cervical lymphadenopathy. Whereas papillary thyroid carcinoma (PTC), the most common type of thyroid carcinoma, has high differentiation and good prognosis, 30%–80% of patients experience cervical lymph node metastasis, which increases the chances of recurrence. Therefore, early identification of cervical lymph node metastasis in PTC is essential for proper clinical treatment (1–4). Besides, China still carries a high burden of tuberculosis. Lymph node tuberculosis occurs when lymph nodes are infected with *Mycobacterium tuberculosis*, which is the most common extrapulmonary tuberculosis. In addition, the increase in immunodeficiency patients exacerbates the spread of tuberculosis, especially in non-Asian regions (5, 6). However, early and proper diagnosis of lymph node disease to inform appropriate treatment strategy remains a challenge (7).

Ultrasonography is the primary diagnostic tool for superficial lymph node lesions. Conventional ultrasound technologies can identify benign and malignant lymph nodes by characterizing lymph node morphology, internal structure, lymphatic portal, or blood flow distribution and can guide lymph node tissue biopsy (8, 9). However, the overlap of benign and malignant lymph nodes on gray-scale sonogram and the inability to display small blood vessels with low flow rate and disturbance by vascular pulsation limit routine ultrasound diagnosis (10, 11).

On the other hand, contrast-enhanced ultrasonography (CEUS) involves an intravenous microbubble contrast agent that uses real-time gray-scale harmonic imaging. It is a new ultrasonic diagnostic technique for normal and enhanced lesions, which can effectively evaluate intratissue perfusion and microcirculation. Previous studies have shown that the CEUS yields superior results in the diagnosis of cervical benign lymph node diseases and tumor lymph node metastasis, with higher accuracy compared with the conventional ultrasound (12, 13). However, data on the use of CEUS in differential diagnosis of cervical lymph node metastasis from PTC and tuberculous lymphadenitis remain scant.

Here, we compared the use of CEUS in characterizing cervical lymph node metastasis from PTC and tuberculous lymphadenitis to provide more data that would aid differential diagnosis.

## Materials and Methods

### Patients

This study was reviewed and approved by the Medical Ethics Committee of Affiliated Hangzhou Chest Hospital of Zhejiang University, and patients gave informed consent. From August 2018 to August 2020, a total of 102 patients who had enlarged cervical lymph nodes at the Affiliated Hangzhou Chest Hospital, Zhejiang University School of Medicine were examined. The pathological data of the lymph nodes of the patients were obtained by core needle biopsy under ultrasound. We included patients who underwent US and CEUS, those with complete medical information, and those without distant metastasis. Patients with severe cardiopulmonary dysfunction; those subjected to tumor surgery, neoadjuvant chemotherapy, or radiotherapy; and those with contraindications for CEUS were excluded from the study.

### US and CEUS Examination

Philips ultrasonic diagnostic instrument (iu22, Philips Healthcare, Bothell) and high-frequency linear array probe (L9-3, frequency 3–9 MHz) were used for patient examinations. The patients took a supine position with a fully exposed neck. The cervical lymph nodes were then scanned. The location of lymph nodes was recorded following the lymph node division established by the American Joint Committee on Cancer Classification (14). Ultrasound characteristic data of the lymph nodes were recorded on the maximum longitudinal and transverse sections such as lymph node size, shape, margin, internal echo, cystic necrosis, calcification, and lymph hilum. Besides, blood flow types of the lymph nodes were recorded using color Doppler ultrasound imaging (CDI), which was mainly divided into avascular, peripheral, hilar, and mixed (15).

CEUS examination low mechanical index (0.06) pulse reverse harmonic imaging and the second-generation sulfur hexafluoride microbubble ultrasonic contrast agent SonoVue (Milan, Italy, Bracco SpA) were used for patient examination. Briefly, the elbow vein was injected with 2.4 ml, followed by 5 ml saline flushing of the pipe (16). Then, there was dynamic observation of lymph node enhancement followed by continuous observation for 2 min. The images were stored in the instrument hard disk for subsequent analysis (7).

The CEUS patterns of the lymph nodes such as enhancement direction, enhancement type, enhancement range, perfusion defect, and pulse-like enhancement were analyzed offline. Enhancement direction was mainly directional centripetal or centrifugal perfusion enhancement. Centripetal perfusion enhancement referred to the filling of the contrast agent from the periphery to the center, while centrifugal perfusion enhancement referred to divergent perfusion from one point to centrifugation. Enhancement type was classified as homogeneous, rim-like enhancement, separated-like enhancement, or asynchronous. Homogeneous enhancement was defined as simultaneous arrival of contrast agents in different parts of the same lymph node. Rim-like enhancement referred to the edge and surrounding, while separated-like enhancement is defined as the internal rendering into a partition or honeycomb sample. On the other hand, asynchronous perfusion referred to the simultaneous enhancement intensity of contrast agents in different parts, where some regions show rapid super enhancement, while others become slow with low enhancement. Enhancement range referred to the traditional ultrasound lymph node size at the peak enhancement. Perfusion defect referred to the absence of contrast agent perfusion in lymph nodes at peak, while pulse-like enhancement was defined as enhancement with pulse synchronization in the arterial phase.

Thereafter, a dedicated software equipped with an ultrasound diagnostic instrument (QLAB, Philips Healthcare) was used to analyze images, while lymph nodes were wrapped around the region of interest to obtain contrast-enhanced time intensity curves (TIC) and blood perfusion parameters. Quantitative parameters included peak intensity (PI), area under the curve (AUC), and time-to-peak intensity (TTP).

Conventional ultrasound and CEUS enhancement patterns were determined by two radiologists with 10 years of experience in diagnosis of lymph node diseases who were blinded on the pathological results. The two radiologists independently diagnosed and analyzed the data, and then finally unified the results after discussion.

### Statistical Analysis

The data were analyzed by SPSS 23.0 statistical software (USA, IBM). Measurement data were analyzed by *t*-test. On the other hand, counting data of the difference between routine ultrasound and contrast-enhanced mode of the lymph nodes of different pathological types were analyzed by *χ* ^2^ test and Fisher accurate test. A *P <*0.05 was taken to be statistically significant. The corresponding sensitivity, specificity, accuracy, and positive or negative predictive value were calculated by comparing the results of lymph node metastasis and tuberculous lymphadenitis with the standard pathological diagnosis.

## Results

### Patients and Study Design

Our analysis showed that 50 patients were male, while 52 were female, with ages ranging from 18 to 77 (mean, 44 ± 4.5) years. All the results were confirmed by puncture pathology. The pathologic results showed that out of the 102 enlarged lymph nodes, 48 were lymph node metastasis of PTC, while 54 were tuberculous lymphadenitis.

### US Examination

**Figure 1:**
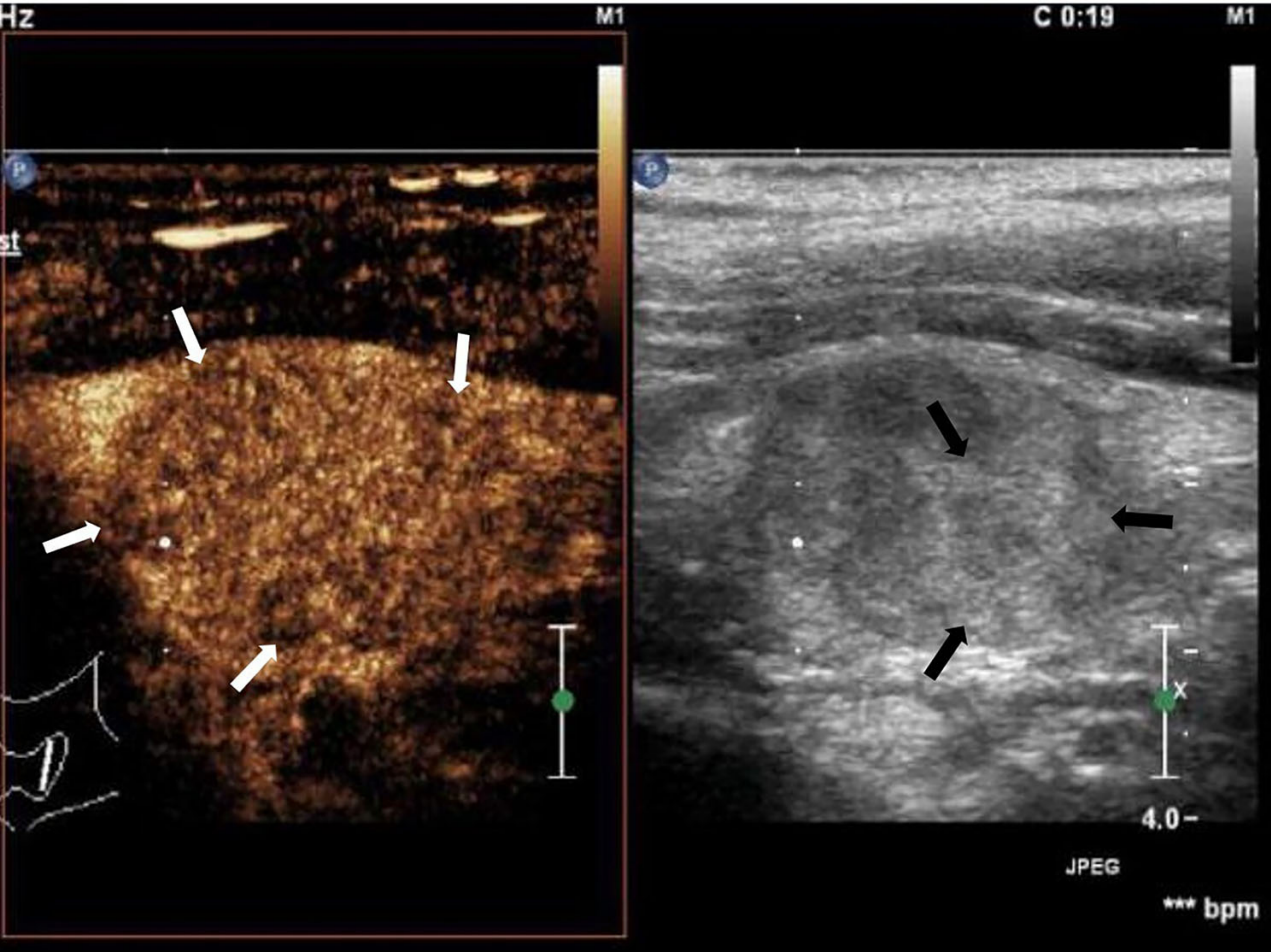
Cervical lymph node metastasis of papillary thyroid carcinoma: lymph node enlargement, absent hilum, heterogeneous, hyperechoic islands (black arrow). Asynchronous enhancement in arterial phase of lymph nodes (white arrow).

Conventional ultrasonography showed no significant differences in the L/S ratio, absent hilum, cystic necrosis, sharpness, calcification, and distribution of blood flow between the two groups. However, hyperechoic islands (**Figure 1**) were found in 43.7% of the lymph node metastasis and in only 22.2% of the tuberculous lymphadenitis samples (*P* < 0.05) (**Table 1**).

**Table 1:** Comparison of ultrasonography features in tuberculous lymphadenitis and lymph node metastasis.

Patterns	Lymph node metastasis	Tuberculous lymphadenitis	*P*
	Nodes (*n* = 48)	Nodes (*n* = 54)	
L/S			
≥2	16 (33.3%)	17 (31.5%)	0.842
<2	32 (66.7%)	37 (68.5%)	
Homogenicity			
Homogeneous	11 (22.9%)	13 (24.1%)	0.891
Heterogeneous	37 (77.1%)	41 (75.9%)	
Echogenicity			
Hypoechoic	29 (60.4%)	31 (57.4%)	0.950
Isoechoic	11 (22.9%)	13 (24.1%)	
Hyperechoic	8 (16.7%)	10 (18.5%)	
Hilum			
Present	10 (20.8%)	16 (29.6%)	0.309
Absent	38 (79.2%)	38 (70.4%)	
Cystic necrosis			
Present	37 (77.1%)	39 (72.2%)	0.574
Absent	11 (22.9%)	15 (27.8%)	
Calcification			
Present	20 (41.7%)	14 (25.9%)	0.092
Absent	28 (58.3%)	40 (74.1%)	
Border			
Sharp	26 (54.2%)	35 (64.8%)	0.274
Indistinct	22 (45.8%)	19 (35.2%)	
Hyperechoic islands			
Present	21 (43.7%)	12 (22.2%)	0.020
Absent	27 (56.3%)	42 (77.8%)	
Vascularity patterns			
Hilar	4 (8.3%)	7 (13.0%)	0.594
Peripheral	22 (45.8%)	18 (33.3%)	
Mixed	19 (39.6%)	24 (44.4%)	
Avascular	3 (6.3%)	5 (9.3%)	

P-value < 0.05 was considered to indicate significance.

### CEUS Examination

**Figure 2:**
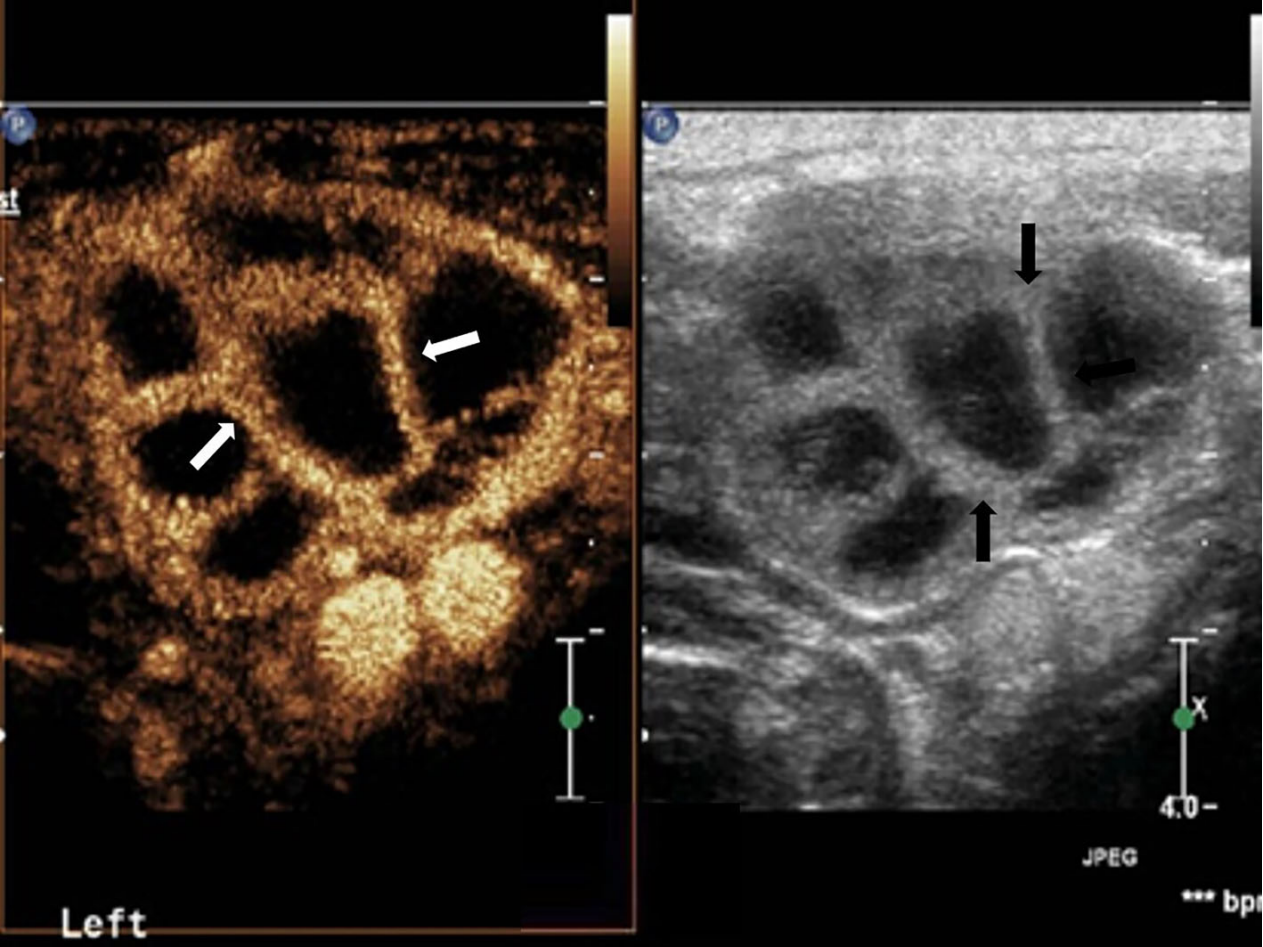
Cervical tuberculous lymphadenitis: lymph node enlargement, absent hilum, honeycomb-like hypoechoic is inside (black arrow). Separated-like enhancement in the arterial phase of lymph nodes (white arrow).

**Figure 3:**
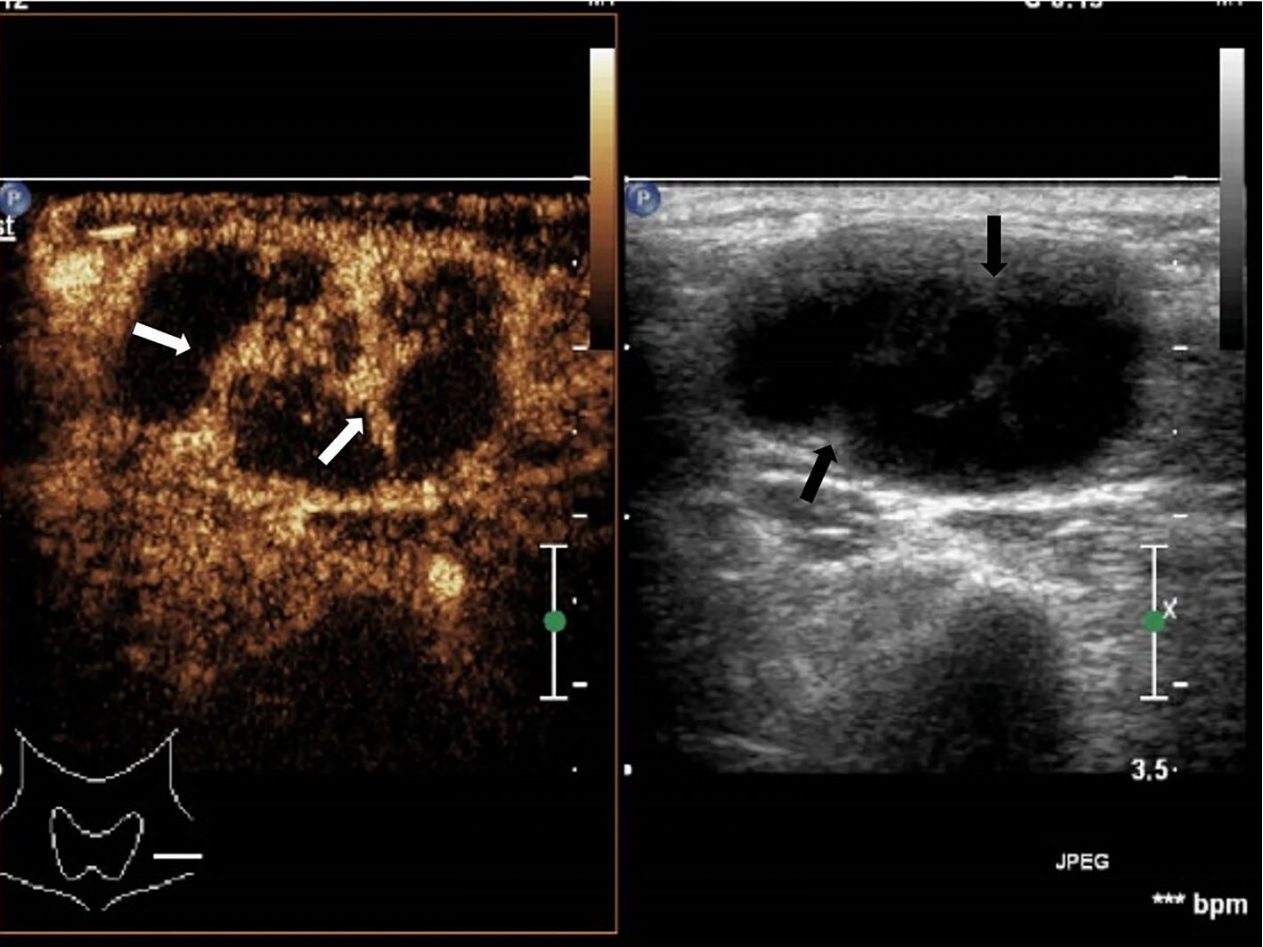
Cervical tuberculous lymphadenitis: lymph node enlargement, absent hilum, honeycomb-like hypoechoic is not significant (black arrow), but CEUS can clearly show enhancement (white arrow).

The CEUS data showed that 31.5% of the cases with separated-like enhancement were in the tuberculous lymphadenitis while only 14.6% of the cases were lymph node metastasis (**Figure 2**). Asynchronous enhancement was found in 64.5% and 35.2% of the patients with lymph node metastasis and tuberculous lymphadenitis, respectively, a feature that was not always clearly shown with conventional US (**Figure 3**). Pulse-like enhancement was found in 41.7% of the lymph node metastasis and in only 20.4% of the tuberculous lymphadenitis (*P* < 0.05). However, there were no statistically significant differences between enhancement direction, enlarged range on CEUS, and perfusion defect (*P* > 0.05), as well as quantitative parameters such as PI, TTP, and AUC between the two groups (**Table 2**).

**Table 2:** CEUS and TIC characteristics of tuberculous lymphadenitis and lymph node metastasis.

Patterns	Lymph node metastasis	Tuberculous lymphadenitis	*P*
	Nodes (*n* = 48)	Nodes (*n* = 54)	
Enhancement direction			
Centripetal	28 (58.3%)	24 (44.4%)	0.161
Centrifugal	20 (41.7%)	30 (55.6%)	
Enhancement type			
Homogeneous	7 (14.6%)	11 (20.4%)	0.027
Rim-like enhancement	3 (6.2%)	7 (13.0%)	
Separated-like enhancement	7 (14.6%)	17 (31.5%)	
Asynchronous	31 (64.6%)	19 (35.1%)	
Enlarged range on CEUS			
Present	22 (45.8%)	27 (50.0%)	0.875
Absent	26 (54.2%)	27 (50.0%)	
Perfusion defect			
Present	39 (81.3%)	42 (77.8%)	0.665
Absent	9 (18.8%)	12 (22.2%)	
Pulse-like enhancement			
Present	20 (41.7%)	11 (20.4%)	0.020
Absent	28 (58.3%)	43 (79.6%)	
TIC			
PI	11.47 ± 3.94	10.69 ± 5.42	0.121
TTP	16.93 ± 5.75	17.78 ± 9.37	0.267
AUC	810.49 ± 420.33	791.37 ± 530.46	0.068

P-value < 0.05 was considered to indicate significance.

CEUS, contrast-enhanced ultrasonography; TIC, time intensity curve; PI, peak intensity; TTP, time-to-peak intensity; AUC, area under the curve.

### Data Analysis

Our analysis showed high specificity of hyperechoic islands (77.8%) and pulse-like enhancement (79.6%), but not sensitivity (43.8%, 41.7%) in the diagnosis of LNM. In addition, asynchronous sensitivity and specificity were 64.6% and 64.8%, respectively (**Table 3**).

**Table 3:** Diagnostic accuracy of US and CEUS for tuberculous lymphadenitis and lymph node metastasis.

Characteristic	Sensitivity (%)	Specificity (%)	Positive predictive value (%)	Negative predictive value (%)	Accuracy (%)
Hyperechoic islands	43.8%	77.8%	63.6%	60.9%	61.8%
Asynchronous	64.6%	64.8%	62.0%	67.3%	64.7%
Pulse-like enhancement	41.7%	79.6%	64.5%	60.6%	61.8%

US, ultrasonography; CEUS, contrast-enhanced ultrasonography.

## Discussion

Ultrasonic features of lymph node tuberculosis overlap with lymph node metastasis which makes it difficult for differential diagnosis using conventional ultrasound (17). Previous studies have demonstrated the ability of CEUS to perform differential diagnosis of benign and malignant lymph nodes. In this study, the separated-like enhancement, pulse-like enhancement, and asynchronous enhancement features presented by the CEUS could help differentiate lymph node tuberculosis from lymph node metastasis. In contrast, the data from perfusion defect, enlarged range on CEUS, enhancement direction, and TIC parameter analyses did not show significant differences.

Conventional US showed no significant differences between tuberculosis and lymph node metastasis, except for the hyperechoic islands within the lymph node metastasis. Clinical physical examination alone cannot distinguish between cervical tuberculous lymphadenitis and cervical PTC metastasis (15, 18). Conventional ultrasound examination has been widely used in the diagnosis of superficial lymph nodes, which characterizes lymph node morphology and internal structure as well as the blood flow distribution within lymph nodes, and metastatic lymph nodes often appear as microcalcification and liquefied necrosis (8, 19). In our study, tuberculous lymphadenitis and lymph node metastasis of the PTC showed some commonality in conventional ultrasound. There were no statistically significant differences in the L/S, hilar, sharp, calcification, necrosis, and color Doppler flow imaging. In addition, the hyperechoic islands in the lymph nodes had a specificity of 77.8% in distinguishing lymph node metastasis from benign lymph nodes. The hyperechoic islands were more frequent in metastatic lymph nodes, which is the result of colonization and aggregation of tumor cells into lymph nodes through lymphatic channels (20). Studies have demonstrated that hyperechogenic islands in lymph nodes are more common in cervical lymph node metastasis of PTC, while lymph node metastasis in other tumors (including other types of thyroid cancer) is not a common occurrence. The hyperechogenic islands are an aggregation of thyroid globulin. Since PTC is a highly differentiated tumor, its tumor cells synthesize thyroglobulin like normal thyroid cells. However, the tumor cells in lymph nodes lack complete follicular structure; thus, they gather into clusters and form hyperechoic, which is hardly seen in lymph node tuberculosis (21, 22).

However, conventional ultrasound is limited by the fact that it does not fully characterize the morphology of the lymph node metastasis, coupled with the fact that not all lymph node metastases have specific and unique features. Similarly, color Doppler ultrasound imaging does not effectively detect low velocity blood flow and cannot comprehensively evaluate the internal structure of the lymph nodes (23).

Tuberculous lymphadenitis presents rim-like enhancement with edge and surrounding coupled with separated-like enhancement. Compared with conventional ultrasound, CEUS can detect tissue necrosis with higher sensitivity, showing no contrast agent perfusion area (24). However, there was the presence of separated-like enhancement in tuberculous lymphadenitis, but not in PTC lymph node metastasis. The separated-like enhancement is associated with rich blood supply status of the lymph node margins and peripheral areas. It stems from the accumulation of *M. tuberculosis* in the lymph node tissues of the hilum which then destroys the normal vascular structure when caseous or liquefactive necrosis occurs, resulting into the lack of blood supply in the central lymph node area. There are many granulated tissues at the edge of the intact lymph node, which is rich in new capillaries. The formation of granuloma in the marginal region of the lymph nodes can induce an immune response in the surrounding soft tissues, which could cause telangiectasia.

Compared with tuberculous lymphadenitis, there was significantly higher asynchronous and pulse-like enhancement in the lymph node metastasis. During the dynamic enhancement process, the contrast agent microbubbles enter the lymph nodes and reach the peak. Besides, the lymph node metastasis of the tumor could be enhanced with the pulse synchronous pulsation, which is rare in tuberculous lymphadenitis. This phenomenon might be associated with the lack of muscle layer and vascular resistance in neovascularization.

Whereas CEUS parameters could provide detailed diagnostic information, there was no significant difference in our study. Previous studies have shown that the *k* value and PI value of tuberculous lymph nodes indicate obvious lymph node metastasis (25). Lymph nodes have a huge supply of blood vessels. When the capillaries of the lesion open, the total number of microbubbles and the PI value increase. The tumor metastasizes to the lymph node, which then makes the blood flow relatively insufficient, thus destroying its normal structure. However, the benign lymph node does not have this manifestation. Other studies have shown that the parameters of malignant lymph nodes such as the PI, TTP, and AUC in patients with nasopharyngeal carcinoma were lower than those of benign lymph nodes, which might be caused by vascular compression by the tumor tissue, thus leading to reduced blood flow in some metastatic lesions (26). Previous research showed significant differences between benign lymph nodes and lymph node metastasis in TTP and AUC. This was associated with the fact that benign lymph node perfusion enhancement time is longer, with low clearance speed, resulting into greater AUC compared with lymph node metastasis (16). In addition, other previous studies have shown inconsistency in the CEUS parameters of benign and malignant lymph nodes, while others have shown no significant difference in the CEUS parameters (PI, TTP, and AUC) between benign and malignant lymph nodes (27). In this study, TTP, PI, and AUC showed no significant difference between tuberculous lymphadenitis and lymph node metastasis. CEUS of the lymph nodes might be affected by the degree of tissue vasodilation or by other factors such as specific contrast agents, scanner parameter adjustment, and patient metabolism. Thus, to obtain more reliable results, there is a need for studies with larger sample sizes.

In this study, enhancement direction, enlarged range on CEUS, and perfusion defects were also associated with tuberculous lymphadenitis, which are not unique to malignant lymph nodes. Previous studies have shown that enhancement direction, enlarged range on CEUS, and perfusion defect define malignant lymph nodes (28). Tumor cells invade lymph nodes and localize in the peripheral parts of lymph nodes. Tumor cell proliferation-induced neovascular abnormalities are prone to poor supply, thus causing tissue necrosis, and thus, contrast agent microbubbles do not reach the site of necrosis, showing uneven enhancement (27, 29). After entering the lymph nodes, *M. tuberculosis* is phagocytosed by macrophages to induce cell-mediated immune response and delayed allergy, macrophage proliferation, and lesion limitation, as well as characteristic tuberculous granuloma and caseous necrosis of cells, resulting in tissue damage (25, 30). The pathophysiology of benign lymph nodes, such as reactive lymph node hyperplasia, is very different from tuberculous lymphadenitis and their CEUS features can not be classified into one category (31).

The CEUS is more sensitive and accurate in the detection of low-speed blood flow compared with conventional ultrasound; thus, it can observe dynamic perfusion processes (7). In our study, pulse-like enhancement and asynchronous enhancement differences were statistically significant in both tuberculous lymphadenitis and lymph node metastasis (*P* < 0.05), but their diagnostic sensitivity and specificity were not satisfactory. The feature of pulse-like enhancement was very specific (79.6%), but not sensitive (41.7%) in the diagnosis of lymph node metastasis. The sensitivity and specificity of asynchronous enhancement were 64.6% and 64.8%, respectively. Thus, the potential of CEUS needs further evaluation.

Whereas our study highlighted important CEUS features, there was no information on the thyroid diagnosis of the patients and no history of tuberculosis, and the patients with ruptured neck or skin color changes were not included in this study. Besides, there was limited sample size, with no detailed classification of lymph nodes.

## Conclusion

Taken together, our study showed that CEUS could provide valuable information on lymph node blood flow, which could help identify both tuberculous lymphadenitis and lymph node metastasis of PTC. However, CEUS application in lymph node diagnosis needs further evaluation.

## Data Availability Statement

The raw data supporting the conclusions of this article will be made available by the authors, without undue reservation.

## Ethics Statement

The studies involving human participants were reviewed and approved by The Ethics Committee of the Affiliated Hangzhou Chest Hospital of Zhejiang University School of Medicine. The patients/participants provided their written informed consent to participate in this study.

## Author Contributions

XZ was responsible for project administration, methodology, data creation, and writing—review and editing. LW, NF, TN, and WT were responsible for data curation and paper revision. All authors contributed to the article and approved the submitted version.

## Funding

This work was supported by the Hangzhou Agriculture and Social Development Research Project (20190101A09), Hangzhou Social Development Project for Social Development (20180533B68), and Hangzhou Science and Technology Plan Guidance Project (20201231Y033).

## Conflict of Interest

The authors declare that the research was conducted in the absence of any commercial or financial relationships that could be construed as a potential conflict of interest.

## Publisher’s Note

All claims expressed in this article are solely those of the authors and do not necessarily represent those of their affiliated organizations, or those of the publisher, the editors and the reviewers. Any product that may be evaluated in this article, or claim that may be made by its manufacturer, is not guaranteed or endorsed by the publisher.
